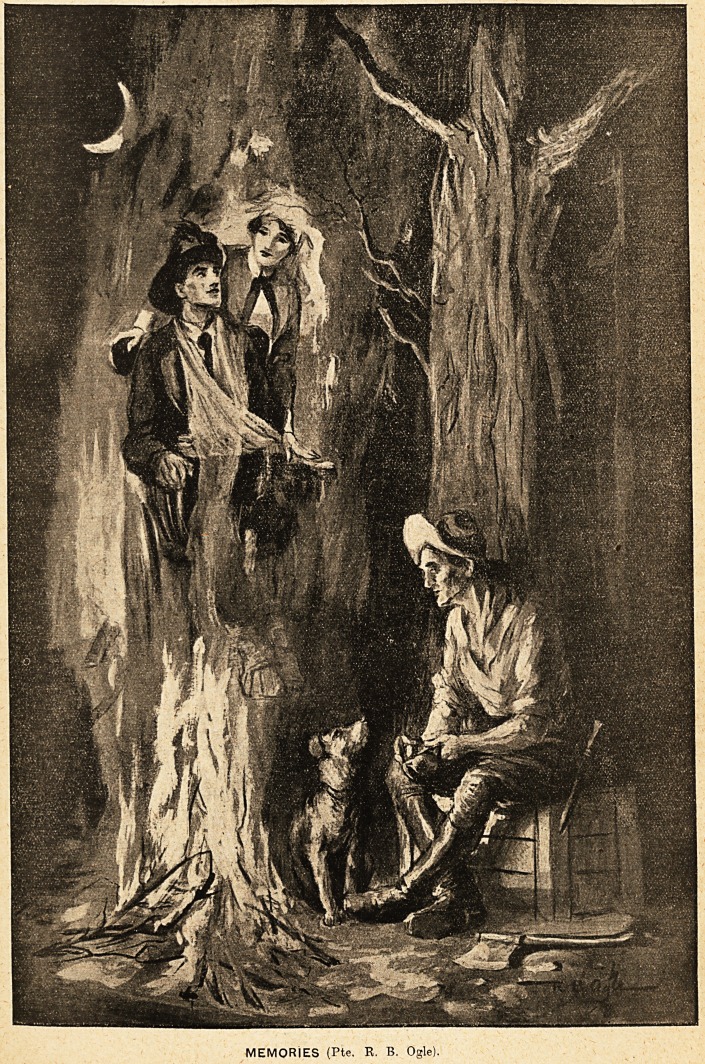# When Wounded—And Afterward

**Published:** 1917-12-15

**Authors:** 


					December 15, 1917. THE HOSPITAL 225
WHEN WOUNDED?AND AFTERWARDS.
I tf
MEMORIES (Pte. R, B. Ogle).

				

## Figures and Tables

**Figure f1:**